# Risk-Based Approach for Defining Retest Dates for Active Pharmaceutical Ingredients and Excipients

**DOI:** 10.3390/ph17070903

**Published:** 2024-07-07

**Authors:** Naseem A. Charoo, Omotayo Akanji, Ziyaur Rahman, Aqeel A. Khan, Aqal Badshah

**Affiliations:** 1 Aramed, 216, Laboratory Complex, Dubai Science Park, Dubai P.O. Box 478861, United Arab Emirates; charoo.naseem@gmail.com; 2 Katchey Laboratories, 26, Adeniyi, Adeniyi Jones Ave, Ogba, Ikeja 101233, Nigeria; omotayoakanji@yahoo.com; 3 Irma Lerma Rangel College of Pharmacy, Texas A&M Health Science Center, Texas A&M University, College Station, TX 77843, USA; 4 Adcan Pharma LLC, ICAD, Abu Dhabi P.O. Box 9824, United Arab Emirates; aqeel.khan@adcanpharma.ae (A.A.K.); aqal.badshah@adcanpharma.ae (A.B.)

**Keywords:** retest, risk assessment, critical material attributes, critical quality attributes, shelf life

## Abstract

Drug substances and excipients must be stored in recommended storage conditions and should comply with their specifications during the retest period for their use in the manufacture of drug products. The ICH (International Council for Harmonization of Technical Requirements for Pharmaceuticals for Human Use) and WHO (World Health Organization) regulatory guidelines mandate that after the retest period, the drug substances must be retested for compliance with the specification and then used immediately in the manufacture of the finished product. Although these substances can be retested multiple times, an emphasis is placed on immediate use following a retest and compliance with standards. The phrase “used immediately” is ambiguous and is left for interpretation. In this article, we will look at the various processes that must be completed to determine the retest date. In addition, we present a risk-based method for establishing retest dates and the time during which material can be used.

## 1. Introduction

The ICH (International Council for Harmonization of Technical Requirements for Pharmaceuticals for Human Use) Q1A guidance document and the WHO (World Health Organization) expert committee report defines the retest period as follows: “The period of time during which the drug substance is expected to remain within its specification and, therefore, can be used in the manufacture of a given drug product, provided that the drug substance has been stored under the defined conditions. After this period, a batch of drug substance destined for use in the manufacture of a drug product should be retested for compliance with the specification and then used immediately. A batch of drug substance can be re-tested multiple times and a different portion of the batch used after each retest, as long as it continues to comply with the specification. For most biotechnological/biological substances known to be labile, it is more appropriate to establish a shelf life than a retest period. The same may be true for certain antibiotics” [1,2]. The guidelines place emphasis on immediate use after a retest and compliance with the specifications. However, “used immediately” is left for interpretation. To define “used immediately”, the WHO expert group has offered some guidance; for example, it stated that the API (active pharmaceutical ingredient) may be utilized within a month of retesting and complying with the specifications [2]. However, the WHO report does not allow the material to be given an additional period (next retest date) equal to the duration set for the retest (first retest date). However, it does allow for repeated retests of the material and its continuing use (as long as specifications are followed).

Similarly, the ICH Q1E guideline allows for data extrapolation to extend the retest period or shelf life beyond what is covered by the long-term data. However, any retest period or shelf life determined based on extrapolation must be verified with additional long-term stability data as soon as possible [3]. Whether or not stability data should be extrapolated depends on how well the change pattern is understood, how well any mathematical model fits the data, and whether or not there is adequate supporting data. Extrapolation should be conducted such that the extended retest period or shelf life is valid for a future batch released with test results that are near to the release acceptance requirements. The guideline offers an illustration of how to assess stability data. It recommends examining quantitative attributes like assay and related substances to find the earliest period when the 95% confidence limit for the mean intersects with the proposed acceptance criterion. The retest period or the shelf-life estimation depends on whether significant change takes place at accelerated conditions or intermediate conditions. For instance, if there is no significant change at accelerated conditions within 6 months and accelerated and long-term data show little or no change over time and little or no variability, statistical analysis is not required and a retest period or shelf life of 2 × period covered by long-term data (not exceeding the period covered by long-term data + 12 months) can be assigned for products stored at room temperature [3]. 

The importance of handling and storing raw materials in a way that prevents degradation, contamination, and cross-contamination cannot be overstated. Moreover, the storage conditions should prevent any physical changes to the material. The principal causes of product degradation and loss of effectiveness are temperature and humidity exposure. The accumulation of static charges in materials held at less than 45% relative humidity might cause the items to dry out, crumble, or stick together, causing issues during tablet pressing and packing [4,5]. Similarly, high-humidity storage environments can compromise potency and effectiveness, leading to material degradation and microbial proliferation. Materials held in fiber drums, bags, or boxes should be kept off the floor and spaced appropriately to allow for cleaning and inspection. The storage conditions and the storage period should be such that they do not negatively impact the quality. There should be sufficient control mechanisms in place to enable FIFO (first in, first out). 

**Figure 1:**
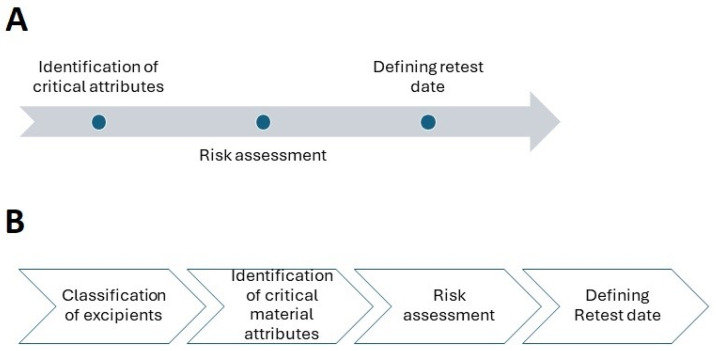
Proposed steps in establishing a retest date: (**A**) active pharmaceutical ingredients; (**B**) excipients.

According to ICH Q7, materials should be reevaluated as needed to determine whether they are suitable for their intended application, especially after prolonged storage or exposure to heat or humidity. Initial API expiry or retest dates can be determined using pilot-scale batches as long as they are manufactured using the same procedure as commercial batches and have the same API quality [6]. In this article, we will look at the many steps that must be taken to establish the retest date. These are depicted in Figure 1. Considering their physicochemical characteristics and the role they play in pharmaceutical formulation, we address these steps independently for APIs and excipients. Furthermore, we propose a risk-based method for determining retest dates.

## 2. Establishing Retest Date for APIs

### 2.1. Identification of Critical Attributes

The critical material attributes are the physical, chemical, biological, or microbiological attributes of the drug substances, which must be within an appropriate range in order for the product to comply with its critical quality attributes (CQAs) [7,8]. 

The critical material attributes that pose a risk to the CQAs of the product during storage or shelf life include particle size, shape, assay, impurity profile, solubility, stability, purity, moisture, polymorphic form, microbial quality, etc. [9,10,11]. 

#### 2.1.1. Particle Size and Shape

Particle size, in relation to surface area, has a substantial impact on the physical and chemical properties of APIs. It plays a pivotal role in defining APIs’ bulk density, flow characteristics, packing features, and cohesion and adhesion characteristics. Its impact on drug solubility, dissolution, bioavailability, content homogeneity, and chemical and physical stability is well recognized [12,13,14]. The dissolution of BCS (Biopharmaceutics Classification System) class II and IV drugs, in particular, is sensitive to the particle size [15,16]. Particle size can also affect manufacturing unit processes such as mixing, granulation, compression, and filling. To achieve uniform mixing, a blend with a narrow particle size distribution is preferred [12,13]. Particles below 100 µm possess interparticulate forces that reduce their segregation tendency under the influence of gravitational forces [17]. The effects of larger particle sizes are more noticeable in powders with narrower particle size distributions [18,19]. There is a significant correlation between the variability of dosage form weight and the flow characteristics, which are influenced by the size and shape of the particles [20]. Direct compression formulations are more prone to segregation and flow disruptions from large particle size variations. The distribution of free-flowing particles’ sizes is also crucial for tablet tensile strength [21]. 

Like particle size, particle shape influences power flow, tensile strength, and blend homogeneity [22,23,24]. The above-listed characteristics may be impacted by any appreciable alteration in particle size, particle size distribution, or shape during storage. It has been demonstrated that extended storage can alter a drug’s particle size, which can have a detrimental effect on content homogeneity [22]. The effect is especially important in the case of products with low API to excipient ratio.

#### 2.1.2. Assay, Impurity Profile, and Stability

**Figure 2:**
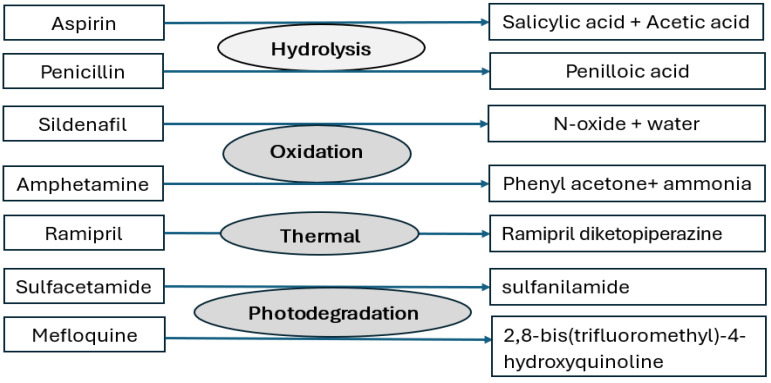
Main degradation mechanisms of some drug substances.

The term “pharmaceutical stability” refers to the ability of an API to withstand changes in quality attributes under specified environmental conditions, such as humidity and temperature [1]. API’s molecular structure and the environment affect how stable it is chemically. Drug substances may degrade due to thermal, hydrolytic, oxidative, or photochemical reactions [25] (Figure 2). Carboxylate ester, amide, and carbamate functional groups in API molecules render them more susceptible to hydrolysis. Similarly, APIs with carboxylic acid, carboxylate ester, hydroxyl group, unsaturated hydrocarbons, amide group, and amine group are prone to oxidation, whereas photolysis is common in carbonyl, nitro, alkene, and aromatic groups containing APIs [26]. Degradation can cause a decrease in the concentration of active substances as well as the generation of undesirable degradation products (impurities). Typically, drug degradation kinetics follow, zero, first, or second-order kinetics. The rate of these degradation reactions is described by a variety of parameters, including the rate constant, activation energy, half-life, and so on. The degradation kinetics of API are also influenced by the pH of the microenvironment, temperature, and the intensity of light exposure [1]. Stress and real-time stability studies are performed to obtain information about degradation products and the mechanism of their formation [27,28,29]. ICH defines stress testing as “Studies undertaken to elucidate the intrinsic stability of the drug substance [1]. Such testing is part of the development strategy and is normally carried out under more severe conditions than those used for accelerated testing”. In these studies, the incremental effects of temperature (e.g., 50 °C, 60 °C, etc.) and humidity (e.g., ≥75% RH), oxidation, and photolysis are carried out on drug substances. The impact of pH on drug substance hydrolysis is also evaluated. Stress-induced degradation products aid in the establishment of degradation pathways [30].Moreover, these studies also help in developing and validating stability-indicating characteristics of the analytical method. These studies are also useful in estimating the intrinsic stability of drug molecules. The degradation kinetics of an API help in the selection of appropriate storage conditions, the selection of container-closure systems, the selection of an optimal formulation development strategy, the anticipation of drug–excipient interactions, and the prediction of shelf life.

#### 2.1.3. Polymorphic Form and Solubility

The rate of dissolution of a solid dosage form is determined by the drug’s saturation solubility, the concentration of solute in solution at a given time, diffusivity, the surface area of the solid particles in contact with GI (gastro-intestinal) fluids, and the mixing or agitation rate of the surrounding media [31]. The particle size, through its effect on surface area, can have a significant impact on the rate of dissolution and thus the bioavailability, particularly for BCS class II and IV drug candidates [15]. Drug bioavailability can decrease dramatically as a result of polymorphic conversion, particularly in the case of BCS class II and IV drug candidates, with significant implications for clinical performance [32,33]. As a result, it is vital to monitor for the occurrence of potential polymorphic transitions during shelf life to guarantee consistent bioavailability following administration [34,35]. There can be significant differences in drugs’ solubility and rate of dissolution amongst solvates, especially hydrates (Table 1). The physical stability of hydrates and their anhydrous counterparts is significantly influenced by the temperature and/or relative humidity of the storage environment. Hydrates may experience fluctuations in water activity at any point during storage. A hydrate is formed when the water activity is sufficient and reaches a critical value (at higher humidity) for hydration. Similarly, as temperatures rise, the instability of hydrates increases, leading to dehydration during the drying process. Transitions from anhydrous to hydrate, and vice versa, can have a significant impact on dissolution rate and bioavailability [36,37]. Dehydration to a less-hydrated state may increase solubility, but stability decreases. Similarly, transitioning from a lower-level hydration state to a higher hydration state may boost thermodynamic stability but reduce solubility. It is crucial that hydrates remain stable in environments where relative humidity is subject to frequent fluctuations. When the hydration level changes due to variations in relative humidity, hydrates may recrystallize into other forms.

Even though crystalline polymorphic forms possess the same chemical composition, their internal crystal structures are distinct. As a result, their physiochemical and pharmacological properties differ. Metastable polymorphism variants are more soluble and bioavailable than their stable counterparts. However, because of its higher energy level, the metastable form is not physically stable and has the potential to change during storage into a stable form. The presence of seeds from one polymorph in another could hasten the transformation [34]. Since API’s crystallinity affects the compact’s (compressed powder blend) mechanical qualities as well as packing and surface characteristics, polymorphic transformation may also influence these aspects [15,38]. 

Amorphous forms are characterized by their high potential energy, absence of long-range order, and thermodynamic instability. They generally revert to a thermodynamically more stable state at a rate determined by the kinetics of the drug in a solid state. Their molecular mobility exceeds that of the crystalline state, making them more reactive and vulnerable to degradation [39]. They provide better solubility due to their higher thermodynamic energy, but this is offset by their chemical instability and transition into thermodynamically more stable crystalline forms. Lower temperatures cause a slowdown in molecular mobility, which either prevents or slows the recrystallization of the amorphous state [40]. On the other hand, molecule mobility increases above the glass transition temperature, causing the solid to crystallize into a more stable crystalline form [41]. Moisture can function as a plasticizer, increasing the molecular mobility and hence, lowering the glass transition temperature of an amorphous solid [42]. It is important to highlight that amorphous materials are highly hygroscopic, which is one of the primary reasons for their transformation to crystalline states. 

The crystal habit of API wields significant influence on powder flow, blending, and compression properties. API’s stability should be tested under a variety of storage circumstances to ensure that it remains chemically and structurally stable during storage. Moisture absorption can influence particle flow and compressibility and impact the manufacturability of the product. Phase transitions can also cause a decrease in drug potency in the product [43]. 

#### 2.1.4. Microbial Quality

When assessing the risk of microbial proliferation in raw material during storage, the following factors must be considered: the material’s origin (natural or synthetic), whether the manufacturing process is likely to reduce or increase the microbial load, and the likelihood of microorganisms multiplying in the material [44]. Material derived from natural sources is typically contaminated with a variety of Gram-positive bacteria, molds, yeasts, and spore forming bacteria. Gum acacia, tragacanth, agar, powdered rhubarb, and starches, for example, are likely to contain bacteria like *Erwinia* spp., *Lactobacillus* spp., *Pseudomonas* spp., *Bacillus* spp., and *Streptococci* spp., as well as molds like *Cladosporium* spp., *Fusarium* spp. and *Alternaria* spp. [45,46]. When estimating the bioburden, it is important to take into account the quantity of microorganisms on the plant material, as this could potentially be a reflection of the conditions during storage and harvest. Furthermore, some microbial species possess innate antimicrobial characteristics that must be considered during testing. If fungal development on the material is detected, the presence and relevance of fungal toxins must be investigated [47].

Except for inadvertent microbial contamination, synthetic raw materials are often devoid of contamination. Although microorganisms can contaminate any raw material, the potential for growth in synthetic materials is quite low. 

A large majority of microorganisms introduced during pharmaceutical manufacturing come from raw materials [48]. As a result, selecting raw materials of high microbiological purity is critical for preventing contamination in the final product. A crucial factor to take into account is the possibility that the raw material preparation process could raise the levels of contamination. As a result, understanding how raw materials are prepared is critical for determining the complete scope of microbiological testing for each substance. Certain refining procedures, for instance, alter the raw materials’ microbiota [49]. Similarly, drying can increase the concentration of spore-forming bacteria, whereas some solubilization techniques may introduce waterborne microorganisms. 

It is crucial to store raw materials carefully, especially hygroscopic ones, to prevent microorganisms from growing. Natural raw materials with a high microbiological count can be sterilized as long as they remain stable. The moisture content of the environment is an important factor to consider during storage. Mold and yeast populations will proliferate if water activity (Aw) is raised above the minimal level needed for growth during storage [50]. Water activity is defined as the ratio of the vapor pressure of water in a raw material (p) to the vapor pressure of pure water (po) at the same temperature [50,51]. The range of the water activity scale is 0 (bone dry) to 1.0 (clean water). Many bacteria cannot grow below water activity values less than 0.8, and even the most extremophilic fungi (xerophilies) are thought to be incapable of growing at Aw values equal to or less than 0.6 [50,51]. It is important to take precautions to guarantee that dry excipients and active ingredients are stored below these threshold Aw values [52]. As a result, raw materials should be held at a constant temperature to avoid evaporation and condensation. Packaging should also be carefully considered because some packaging configurations, such as unlined paper sacks, may absorb moisture and undergo microbial deterioration, contaminating their contents as a result [49,53].

#### 2.1.5. Nitrosamines

Various formulation components, including drug substances, excipients, processing aids, solvents, and packaging material may lead to the formation of nitrosamines in drug products. Another possible cause of nitrosamine generation is the manufacturing process [54]. The example of ranitidine demonstrates that nitrosamines can also form during shelf life [55,56]. Ranitidine tablets that were about to expire had higher levels of NDMA (N-nitrosodimethylamine) than those that were recently manufactured, indicating that degradation was the reason behind the formation of NDMA. Ranitidine powder and tablet accelerated stability trials also showed that NDMA levels rose well over the FDA’s daily dosage limits. Various factors lead to this degradation, including oxidation, moisture levels, self-decomposition of ranitidine, heat, crystal morphology, etc. [55,56,57,58]. Therefore, products that contain unstable amines should be evaluated in a formal stability program under both real-time and accelerated stability study conditions. Given the elevated carcinogenic potential associated with nitrosamines and their distinct processes of generation during storage, nitrosamine testing must be part of the battery of tests that must be run at retest, particularly for high-risk APIs and excipients.

### 2.2. Risk Assessment 

Risk assessment is “A systematic process of organizing information to support a risk decision to be made within a risk management process. It consists of the identification of hazards and the analysis and evaluation of risks associated with exposure to those hazards” [59]. It is necessary to describe the problem statement, i.e., “what critical material attributes could go wrong”. FMEA (failure mode and effects analysis) and other risk assessment tools can be utilized for this purpose [60]. The likelihood of the risks occurring and their severity in relation to quality are examined. The probability of occurrence is mostly determined from historical data and experience, highlighting the importance of reliable, accessible, and robust data. Risks are ranked, and a probability–impact matrix is created to prioritize them [61,62].

API stability data should be analyzed, as well as critical attributes. Changes in important material attributes should be examined for their impact on product CQAs. Typically, the DMF (drug master file) contains the stability data, as well as other critical information needed to assess the stability of an API. One significant aspect that could significantly reduce the therapeutic efficacy of the drug candidate is the physical and chemical degradation of the API. Therefore, API should remain in a stable state in the dosage form until the expiration date. The method of administration, safety level of degradants, and capability of stabilizing medicine in formulation all contribute to achieving an acceptable level of stability. The most prevalent cause of drug instability is a chemical reaction, which causes drugs’ potency to drop and impurities to increase. Drug compounds are vulnerable to various degradation pathways, depending on their chemical structures. For example, in the gastrointestinal tract, drugs containing functional groups, such as ester, amide, lactum, lactone, and sulphoamides, may undergo hydrolysis, oxidation, and reduction processes that are facilitated by the gut’s pH, enzymes, or bacterial flora. The chemical stability of drug candidates is also affected by other environmental conditions, such as excipients, oxygen, light, temperature, and moisture. To avoid oxidation and pH-mediated hydrolytic degradation, the pH of the formulation microenvironment may need to be controlled using buffer systems. Stress testing is an essential tool for investigating degrading impurities and their pathways. The degradation precursors and mechanism provide critical information for selecting the manufacturing process and appropriate packing material for the product [63].

Based on the physical and chemical properties of an API and its susceptibility to degradation owing to environmental conditions, it can be categorized as high risk, moderate risk, or low risk. As an example, fluconazole is known to show polymorphism. Polymorphic forms I, II, III, and a monohydrate form have been reported. The solubility of monohydrate form, polymorphic form I and polymorphic form II, as reported in the literature, is 4.21, 4.96 and 6.59 mg/mL, respectively [64,65,66]. Similarly, the intrinsic dissolution rate of form I and the monohydrate form is lower than that of form II. Polymorphic form II conversion to other forms is reported to take place rapidly at higher humidities (>84% RH). As a result, monitoring the polymorphic conversion during shelf life would be necessary [64]. The tests that must be conducted during retesting include appearance, loss on drying (or water content), assay, related substances, polymorphic form quantification, and microbiological quality. Until sufficient data is available to prove otherwise, the API will be classified as high risk. On the other hand, the losartan (Form I) API, which is frequently used in finished formulations, is relatively stable; retest dates of up to five years from API manufacturers are not unusual [67]. The following attributes would need to be retested: appearance, water content, assay, related substances, and nitrosamines. The API is likely to be categorized as a low-risk API unless development and stability data have confirmed otherwise. 

### 2.3. Retest

The API should be retested at the defined interval for attributes identified in the risk assessment. Based on the observed change (significant or non-significant change in critical attributes), a new retest period can be assigned. The definition of significant change is defined in the regulatory documents [1,3]. The retest time may be extended if there is no significant change, as opposed to when significant change is observed in critical material attributes. For low-risk APIs, the retest period can be longer than for moderate- and high-risk APIs. Table 2 presents an example for assigning retest dates. Manufacturers can set retest dates based on the available data and company policy. The retest dates shown in Table 2 take into account retest dates subsequent to the retest date specified by the API manufacturer. If the API manufacturer has assigned extended retest dates, it is the responsibility of the finished product manufacturer to retest the material, although the frequency of retest can be greater than given in Table 2 considering that adequate data has been generated by the API manufacturer to support the retest date. 

## 3. Excipients

### 3.1. Classification of Excipients

The first step involves categorizing excipients based on the existing stability data or by evaluating their physical and chemical properties. Based on the stability of the excipients in their commercial package, IPEC (International Pharmaceutical Excipients Council) categorizes excipients into the following general categories: very stable, stable, and limited-stability excipients [68]. 

#### 3.1.1. Very Stable

These excipients have a documented track record of stability in the specified packaging for at least five years. An assessment of their known characteristics can be used to forecast their stability. Furthermore, their manufacturing processes are robust and validated and any modifications in their manufacturing process are unlikely to affect the stability of these excipients. Ongoing stability studies are unnecessary if quality attributes remain stable for ≥60 months, as demonstrated by relevant literature and/or stability studies.

#### 3.1.2. Stable

These excipients have a minimum retest/re-evaluation interval of at least 24 months but less than 60 months. Their stability can be determined by analyzing stability-indicating attributes such as assay and impurities; hence, data must be generated to substantiate the retest/re-evaluation interval and expiration date. The stability of these excipients is supported by sufficient literature citations and/or stability studies. Nevertheless, compared to excipients categorized as extremely stable, their stability is more susceptible to modifications in the manufacturing process or product packaging. 

#### 3.1.3. Limited Stability

They have a retest/re-evaluation interval or expiration date of less than 2 years. Like stable excipients, their primary stability-indicating parameters, including assay and impurities, help to assess their stability characteristics. Quite often, only a limited amount of stability data is available to support the expiration date or the retest or re-evaluation of these excipients. This category of excipients is distinguished by stability characteristics that render them more susceptible to changes in the manufacturing process or product packaging, necessitating specific packaging and storage conditions. These excipients may contain functional groups that are prone to hydrolysis and oxidation. Additionally, these excipients may also be susceptible to moisture absorption, heat or light deterioration, and viscosity change; hence, their compliance with specifications is compromised by unfavorable environmental conditions. Therefore, an on-going stability program (accelerated and real time) is recommended to be conducted in the packaging in which they are to be commercialized. Any changes in the packaging (container-closure system) or the manufacturing process that could impact excipients’ stability would require that a new stability study be performed. However, if moisture vapor penetration or oxygen permeation studies demonstrate that the new package is similar to or superior to the packaging system used for the stability studies, a new stability study may not be necessary to determine the impact on excipient stability. The finished product manufacturer should have access to a summary stability report providing information on the stability conditions, attributes monitored, packaging, and the stability study’s findings. An excipient can be categorized into more than one class depending on how much protection the product packaging offers [68]. 

### 3.2. Identification of Critical Material Attributes and Risk Assessment 

Critical material attributes of excipients must be identified as they have the potential to change during storage or have an impact on the CQAs of the finished products [15]. While performing risk assessment, the impact of the storage condition on material attributes, as well as on product CQAs, should be considered. 

An example of a stable and very stable excipient is provided below for ease of comprehension.

#### 3.2.1. Polyplasdone XL-10 (Crospovidone): Example of Stable Excipient

Crospovidone is a white-to-creamy-white, free-flowing, fine hygroscopic powder. It is a water insoluble cross-linked homopolymer of N-vinyl-2-pyrrolidinone [69]. It is commonly used as a tablet and capsule disintegrant at a concentration of 2–5%. It exhibits high capillary activity and hydration capacity. The particle size of crospovidone may impact the disintegration property of the finished product, with larger particles providing faster disintegration in comparison to smaller particles [15]. The harmonized specifications of crospovidone are presented in Table 3. The Polyplasdone™ XL-10 is a crospovidone brand manufactured by Ashland (Wilmington, DE, USA) [69]. The manufacturer assigns a retest date of 24 months for its brand in specific packaging. Crospovidone has a remarkable impact on the disintegration time and dissolution of the solid dosage forms. Moreover, it is known to contain peroxides, which can trigger the degradation of oxidation-sensitive drug candidates [70]. The dissolution tests are an essential part of the battery of tests for finished-product specifications and can detect any impact that storage conditions may have on the disintegration properties of crospovidone. Drug–excipient compatibility performed during product development can detect any impact on impurity profile characteristics arising from peroxides present in crospovidone [71]. Further, related substances are an important component of the finished-product specifications. Therefore, description, impurity A, loss on drying, peroxides, microbial test, and particle size can be recommended to be performed at retest (Table 3). The excipient manufacturer must also confirm, through stability studies on a reasonable number of batches, that nitrosamine impurities in the raw material are under control [72].

#### 3.2.2. Pearlitol^®^ 200 SD (Mannitol): Example of Very Stable Excipient

Mannitol is a white, odorless, crystalline, free flowing powder or granules. Mannitol is used as a diluent in pharmaceutical preparations [73]. It is not hygroscopic and is thus used with moisture-sensitive drugs. It also possesses negative heat of solution, sweetness, and a good mouth feel due to which it is also used in chewable tablets. The USP (United States Pharmacopoeia), Ph.Eur. (European Pharmacopoeia), and JP (Japanese Pharmacopoeia) have harmonized mannitol monograph. The specifications are shown in Table 4. As it is used in considerably high quantities in the formulation, its particle size can play a significant role in flow, compressibility, and other characteristics [74]. Hence, in-house specifications are typically set by the finished product manufacturers for the particle size. Pearlitol^®^ 200SD is a mannitol brand manufactured by Roquette Pharma (1347 Beaver Channel Parkway, Clinton, IA, USA). The manufacturer has assigned it an expiry date of 5 years [74]. The CQAs that are likely to be impacted due to mannitol include uniformity of dosage units and dissolution. It may also influence the compressibility of the powder blend. However, typical quality control tests for a finished product, such as uniformity of dosage units, disintegration time, and dissolution, would facilitate timely detection of any impact on these parameters. Moreover, since it is a very stable excipient, the occurrence of these events is very low. Therefore, description/appearance, loss on drying, assay, microbial test, related substances, reducing sugars, and particle size tests can be recommended to be performed at retest (Table 4).

#### 3.2.3. Nitrosamine Risk Due to Excipients

The composition and impurity profile of excipients are often determined by their source (natural or synthesized). Nitrosating impurities (nitrites and nitrates) can be present in regularly used excipients, such as polyvinyl pyrrolidone (binding agent), pregelatinized starch (diluent and disintegrant), sodium starch glycolate (disintegrant), cross-polyvinyl pyrrolidone(disintegrant), lactose (diluent), and croscarmellose sodium (disintegrant); therefore, a risk factor in nitrosamine generation exists in pharmaceutical products containing amine containing components [72,75]. These impurities in excipients originate from processing water and manufacturing procedures, particularly those that involve acids and bleaching chemicals. One high-risk event that might lead to the generation of nitrosamines is oxidation during the drying process. Research on metformin tablets has demonstrated that the co-existence of two processing parameters, namely, heat and water, as well as the excipients’ nitrate and nitrite contents, are important factors in the formation of nitrosamine impurities. Various control strategies have been proposed to mitigate the risk of nitrosamine formation in pharmaceutical products, including the risk arising from the presence of nitrites and nitrate impurities in excipients [57,76]. 

### 3.3. Defining Retest Period

Upon receipt, the excipients undergo initial testing. The retest date will be determined by the finished product manufacturer’s policy, the stability of the excipients, and other available information. Manufacturers may choose to retest (first retest date), for example, one year later, at 50% of the excipient manufacturer’s designated retest or shelf life, or on the excipient manufacturer’s assigned retest date (whichever comes first). It is important to note that the excipient cannot be retested after the expiration date specified by the manufacturer. Within the boundaries of the retest date assigned by the excipient manufacturer, the finished product manufacturer may designate a second retest date either 1 year after the first retest date analysis or the excipient manufacturer’s retest date (whichever comes first), subject to the first retest date analytical data. Beyond the retest date designated by the excipient manufacturer, retest dates can be assigned depending on the stability characteristics of the excipients. For instance, 1 year may be assigned to very stable excipients, 6 months to stable excipients, and 3 months to limited-stability excipients (Table 5). Of course, this will be determined based on the analytical results of earlier retest analyses, as well as compliance to specifications.

## 4. Conclusions

Currently, regulatory guidelines mandate that APIs or excipients be used immediately after a retest. The time following a retest during which material can be used is not specified in regulatory documents. We have proposed a risk-based strategy for determining the period following retest dates during which material can be used in pharmaceutical manufacture. A three-step approach for APIs and a four-step approach for excipients are proposed. The emphasis is placed on defining critical material attributes, which will facilitate reducing the number of test attributes requiring retesting. 

## Figures and Tables

**Table 1:** Examples of APIs showing polymorphism.

Drug	Polymorph Used in Formulation	Polymorphic Transformation
Ritonavir	Form I	Conversion of Form I to Form II led to failure in meeting dissolution specification. The two forms differ in solubility significantly.
Rifaximin	Rifaximin-α	Conversion to amorphous rifaximin, may lead to systemic absorption of this otherwise locally acting GIT (gastro-intestinal tract) drug resulting in serious safety issues.
Celiprolol hydrochloride	Form I	Form I converts to form II on exposure to high humidity (>80% RH) over a period of one month. Therefore, it is essential to control humidity condition during its storage and processing.
Carbamazepine	Form III	Form III is unstable and absorbs high percentage of water on storage and converts to carbamazepine dihydrate resulting in drop in dissolution. Form I is relatively stable. The dissolution rate rank orders as III > form I > dihydrate.

**Table 2:** An illustration of retest date assignment based on risk assessment.

Risk Category	Retest Period	
	Non-Significant	Significant
Low	6 months	3 months
Medium	6 months	2 months
High	3 months	1 month

**Table 3:** Specifications of crospovidone and risk-assessment-based retesting.

Parameters	USP *	Ph.Eur. *	JP *	Polyplasdone™ XL-10				
				Stability	Impact on Product CQAs	Occurrence Probability (Change in Material Attributes)	Detection ^@^	Tests to Be Performed at Retest
Definition (description/appearance)	+	+	+	Stable	High Can impact CQAs of product such as disintegration time, dissolution and impurity profile.	Low to moderate	High Control strategy for finished product	+
Assay (Nitrogen)								-
Identification	+	+	+					-
Peroxides	+	+	+					+
Water soluble substances	+	+	+					-
Impurity A	+	-	+					+
Loss on drying	+	+	+					+
Residue on ignition/sulfated ash	+	+	+					-
Assay	+	+	+					+
Storage	+	+	+					-
Microbial enumeration test	-	-	-					-
Particle size	In-house test							+

* USP: United States Pharmacopoeia; Ph.Eur.: European Pharmacopoeia; JP: Japanese Pharmacopoeia. ^@^ The ability to detect change in material attributes on CQAs. + attribute present or test to be performed. - attribute absent or test not to be performed.

**Table 4:** Specifications of mannitol and risk-assessment-based retesting.

Parameters	USP *	Ph.Eur. *	JP *	Pearlitol ^®^				
				Stability	Impact on Product CQAs	Occurrence Probability (Change in Material Attributes)	Detection^@^	Tests to Be Performed at Retest
Definition (description/appearance)	+	+	+	Very stable	High Can impact CQAs of product such as uniformity of dosage units, dissolution and influence compressibility.	Low	High Control strategy for finished product	+
Identification	+	+	+					-
Assay	+	+	+					+
Related substances (impurities)	+	+	+					+
Reducing sugars	+	+	+					+
Nickel	+	-	+					-
Melting range or temperature	+	+	+					-
Appearance of solution	+	+	+					-
Loss on drying	+	+	+					+
Conductivity	+	+	+					-
Microbial enumeration test	+	+	-					+
Bacterial endotoxin test	+	+	-					+
Labeling	+	+	-					-
Particle size	In-house test							+

* USP: United States Pharmacopoeia; Ph.Eur: European Pharmacopoeia; JP Japanese Pharmacopoeia. ^@^ The ability to detect impact of change in material attributes on CQAs. + attribute present or test to be performed. - attribute absent or test not to be performed.

**Table 5:** An illustration of defining retest period for excipients.

Retest Period		
First Retest Date	Second Retest Date	
**Within** the boundaries of the retest date assigned by the excipient manufacturer	**Within** the boundaries of the retest date assigned by the excipient manufacturer	**Beyond** the retest date designated by the excipient manufacturer
One year after receiving and initial analysis At 50% of the manufacturer’s designated retest Manufacturer’s assigned retest date *Whichever comes first among the above* *options*	One year after the first retest date analysis Manufacturer’s assigned retest date *Whichever comes first among the above* *options*	Based on the stability characteristics of the excipients Example: - Very stable: 12 months; - Stable: 6 months; - Limited stability: 3 months.

## References

[1] (2003). ICH Harmonised Tripartite Guideline. Stability Testing of New Drug Substances and Products, Q1A(R2). https://database.ich.org/sites/default/files/Q1A%28R2%29%20Guideline.pdf.

[2] WHO Expert Committee on Specifications for Pharmaceutical Preparations. Fifty-Second Report. https://iris.who.int/bitstream/handle/10665/272452/9789241210195-eng.pdf?sequence=1.

[3] (2003). ICH Harmonised Tripartite Guideline. Evaluation for Stability Data, Q1E. https://database.ich.org/sites/default/files/Q1E_Guideline.pdf.

[4] Deng T., Garg V., Bradley M.S.A. (2023). Electrostatic charging of fine powders and assessment of charge polarity using an inductive charge sensor. Nanomanufacturing.

[5] Peart J. (2001). Powder electrostatics: Theory, techniques and applications. KONA Powder Part. J..

[6] ICH Harmonised Tripartite Guideline. Good Manufacturing Practice Guide for Active Pharmaceutical Ingredients. https://database.ich.org/sites/default/files/Q7%20Guideline.pdf.

[7] (2009). ICH Harmonised Tripartite Guideline. Pharmaceutical Development Q8(R2). https://database.ich.org/sites/default/files/Q8_R2_Guideline.pdf.

[8] Cogdill R.P., Drennen J.K. (2008). Risk-based quality by design (QbD): A Taguchi perspective on the assessment of product quality, and the quantitative linkage of drug product parameters and clinical performance. J. Pharm. Innov..

[9] Somma R. (2007). Development knowledge can increase manufacturingcapability and facilitate quality by design. J. Pharm. Innov..

[10] Yu L.X. (2008). Pharmaceutical quality by design: Product andprocess development, understanding, and control. Pharm. Res..

[11] Lionberger R.A., Lee S.L., Lee L., Raw A., Yu L.X. (2008). Quality by design: Concepts for ANDAs. AAPS J..

[12] Shekunov B.Y., Chattopadhyay P., Tong H.H.Y., Chow A.H.L. (2006). Particle size analysis in pharmaceutics: Principles, methods and applications. Pharm. Res..

[13] Standish N. (1985). Studies of size segregation in filling and emptying a hopper. Powder Technol..

[14] Samadani A., Pradhan A., Kudrolli A. (1999). Size segregation of granular matter in silo discharges. Phys. Rev. E.

[15] Charoo N.A. (2020). Critical excipient attributes relevant to solid dosage formulation manufacturing. J. Pharm. Innov..

[16] Hlinak A.J., Kuriyan K., Morris K.R., Reklaitis G.W., Basu P.K. (2006). Understanding critical material properties for solid dosage form design. J. Pharm. Innov..

[17] Abdullah E.C., Geldart D. (1999). The use of bulk density measurements as flowability indicators. Powder Technol..

[18] Fayed M.E., Otten L. (1997). Handbook of Powder Science & Technology.

[19] Andrews G., Jones D., Zhai H., Diak O.B., Walker G., Gad S.C. (2008). Effects of grinding in pharmaceutical tablet production. Pharmaceutical Manufacturing Handbook.

[20] Osorio J.G., Muzzio F.J. (2013). Effects of powder flow properties on capsule filling weight uniformity. Drug Dev. Ind. Pharm..

[21] Fichtner F., Rasmuson A., Alderborn G. (2005). Particle size distribution and evolution in tablet structure during and after compaction. Int. J. Pharm..

[22] Kaerger J.S., Edge S., Price R. (2004). Influence of particle size and shape on flowability and compactibility of binary mixtures of paracetamol and microcrystalline cellulose. Eur. J. Pharm. Sci..

[23] Shinohara K., Miyata S. (1984). Mechanism of density segregation of particles in filling vessels. Ind. Eng. Chem. Process Des. Dev..

[24] Kuentz M., Leuenberger H. (2000). A new theoretical approach to tabletstrength of a binary mixture consisting of a well and a poorly compactable substance. Eur. J. Pharm. Biopharm..

[25] Rácz I. (1984). Mechanismen der Arzneimittelzersetzung—Arzneistoffstabilisierung [Mechanisms of drug decomposition-drug stabilization]. Pharmazie.

[26] Bhangare D., Rajput N., Jadav T., Sengupta P. (2022). Systematic strategies for degradation kinetic study of pharmaceuticals: An issue of utmost importance concerning current stability analysis practices. J. Anal. Sci. Technol..

[27] Blessy M., Patel R.D., Prajapati P.N., Agrawal Y.K. (2014). Development of forced degradation and stability indicating studies of drugs-A review. J. Pharm. Anal..

[28] Baertschi S.W., Alsante K.M., Reed R.A. (2016). Pharmaceutical Stress Testing: Predicting Drug Degradation.

[29] Qiu F., Scrivens G. (2018). Accelerated Predictive Stability (APS): Fundamentals and Pharmaceutical Industry Practices.

[30] Huynh-Ba K. (2009). Pharmaceutical Stability Testing to Support Global Markets.

[31] Gao Y., Glennon B., He Y., Donnellan P. (2021). Dissolution kinetics of a BCS class II active pharmaceutical ingredient: Diffusion-based model validation and prediction. ACS Omega.

[32] Panda R., Lankalapalli S. (2023). Bbioavailability and polymorphic stability challenges affecting drug product’s potential: A critical evaluation and pertinent solution. Asian J. Pharm. Cli. Res..

[33] Censi R., Di Martino P. (2015). Polymorph Impact on the Bioavailability and Stability of Poorly Soluble Drugs. Molecules.

[34] Charoo N.A., Cristofoletti R., Kim S.K. (2017). Integrating biopharmaceutics risk assessment and in vivo absorption model in formulation development of BCS class I drug using the QbD approach. Drug Dev. Ind. Pharm..

[35] (1999). ICH, Harmonised Tripartite Guideline Specifications: Test Procedures and Acceptance Criteria for New Drug Substances and New Drug Products: Chemical Substances, Q6A. https://database.ich.org/sites/default/files/Q6A%20Guideline.pdf.

[36] Brittain H.G., Grant D.J.R., Myrdal P.B., Brittain H.G. (2009). Effects of Polymorphism and Solid-State Solvation on Solubility and Dissolution Rate. Polymorphism in Pharmaceutical Solids.

[37] Zhou Y., Wang J., Xiao Y., Wang T., Huang X. (2018). The effects of polymorphism on physicochemical properties and pharmacodynamics of solid drugs. Curr. Pharm. Des..

[38] Avicel® for Solid Dosage Forms. http://www.fmcbiopolymer.com/Pharmaceutical/Products/Avicelforsoliddoseforms.aspx.

[39] Rumondor A.C., Taylor L.S. (2010). Effect of polymer hygroscopicity on the phase behavior of amorphous solid dispersions in the presence of moisture. Mol. Pharm..

[40] Talaczynska A., Dzitko J., Cielecka-Piontek J. (2016). Benefits and limitations of polymorphic and amorphous forms of active pharmaceutical ingredients. Curr. Pharm. Des..

[41] Valenti S., Barrio M., Negrier P., Romanini M., Macovez R., Tamarit J.L. (2021). Comparative physical study of three pharmaceutically active benzodiazepine derivatives: Crystalline versus amorphous state and crystallization tendency. Mol. Pharm..

[42] Červinka C., Fulem M. (2021). Structure and glass transition temperature of amorphous dispersions of model pharmaceuticals with nucleobases from molecular dynamics. Pharmaceutics.

[43] Yoshinari T., Forbes R.T., York P., Kawashima Y. (2002). Moisture induced polymorphic transition of mannitol and its morphological transformation. Int. J. Pharm..

[44] Sutton S., Jimenez L. (2012). A Review of reported recalls involving microbiological control 2004-2011 with emphasis on FDA considerations of ‘objectionable organisms. Am. Pharm. Rev..

[45] Dao H., Lakhani P., Police A., Kallakunta V., Ajjarapu S.S., Wu K.W., Ponkshe P., Repka M.A., Narasimha Murthy S. (2018). Microbial stability of pharmaceutical and cosmetic products. AAPS Pharmscitech.

[46] Ratajczak M., Kubicka M.M., Kamińska D., Sawicka P., Długaszewska J. (2015). Microbiological quality of non-sterile pharmaceutical products. Saudi Pharm. J..

[47] Martínez-Bermúdez A., Rodríguez-de Lecea J., Soto-Esteras T., Vázquez-Estévez C., Chena-Cañete C. (1991). Tipos de contaminantes microbianos de materias primas farmacéuticas [Types of microbial contaminants in pharmaceutical raw materials]. Rev. Latinoam. De Microbiol..

[48] Cundell T. (2016). Mold monitoring and control in pharmaceutical manufacturing areas. Am. Pharm. Rev..

[49] Russell M. (1996). Microbial Quality Assurance in Cosmetics, Toiletries and Non-Sterile Pharmaceuticals.

[50] Grant W.D. (2004). Life at low water activity. Philos. Trans. R. Soc. London. Ser. B Biol. Sci..

[51] He Y., Li Y., Salazar J.K., Yang J., Tortorello M.L., Zhang W. (2013). Increased water activity reduces the thermal resistance of salmonella enterica in peanut butter. Appl. Environ. Microbiol..

[52] United States Pharmacopoeia (2012). Microbiological Examination of Non-Sterile Products: Tests for Specified Microorganisms.

[53] Denyer S.P., Baird R.M. (2007). Microbial contamination, spoilage and hazard. Guide to Microbiological Control in Pharmaceuticals and Medical Devices.

[54] Tuesuwan B., Vongsutilers V. (2021). Nitrosamine contamination in pharmaceuticals: Threat, impact, and control. J. Pharm. Sci..

[55] FDA-U.S. Food & Drug Administration (2020). FDA Requests Removal of All Ranitidine Products (Zantac) from the Market. https://www.fda.gov/news-events/press-announcements/fda-requests-removalall-ranitidine-products-zantac-market.

[56] EMA-European Medicines Agency (2019). EMA to Review Ranitidine Medicines following Detection of NDMA. Press Release..

[57] Charoo N.A., Dharani S., Khan M.A., Rahman Z. (2023). Nitroso impurities in drug products: An overview of risk assessment, regulatory milieu, and control strategy. AAPS PharmSciTech.

[58] Ashworth I.W., Dirat O., Teasdale A., Whiting M. (2020). Potential for the formation of N-nitrosamines during the manufacture of active pharmaceutical ingredients: An assessment of the risk posed by trace nitrite in water. Org. Process Res. Dev..

[59] (2023). ICH, Harmonised Guideline. Quality Risk Management Q9(R1). https://database.ich.org/sites/default/files/ICH_Q9%28R1%29_Guideline_Step4_2023_0126_0.pdf.

[60] Stamatis D.H. (2003). Failure Mode and Effect Analysis, Fmea from Theory to Execution.

[61] Charoo N.A., Ali A.A. (2013). Quality risk management in pharmaceutical development. Drug Dev. Ind. Pharm..

[62] Githens G.D., Peterson R.J. Using risk management in the front end of projects. Proceedings of the Project Management Institute 32nd Annual Seminars & Symposium.

[63] Florence A.T., Attwood D. (1998). Drug Stability. Physicochemical Principles of Pharmacy.

[64] Charoo N., Cristofoletti R., Graham A., Lartey P., Abrahamsson B., Groot D.W., Kopp S., Langguth P., Polli J., Shah V.P. (2014). Biowaiver monograph for immediate-release solid oral dosage forms: Fluconazole. J. Pharm. Sci..

[65] Alkhamis K.A., Obaidat A.A., Nuseirat A.F. (2002). Solid-state characterization of fluconazole. Pharm. Dev. Technol..

[66] Park H.J., Kim M.S., Kim J.S., Cho W., Park J., Cha K.H., Kang Y.S., Hwang S.J. (2010). Solid-state carbon NMR characterization and investigation of intrinsic dissolution behavior of fluconazole polymorphs, anhydrate forms I and II. Chem. Pharm. Bull..

[67] Public Assessment Report Scientific Discussion: Losartan kalium Pharmaclan 50 mg and 100 mg Film-Coated Tablets (Losartan Potassium). NL/H/5449/001-002/DC, 2023. https://www.geneesmiddeleninformatiebank.nl/pars/h128848.pdf.

[68] IPEC (2022). Excipient Stability Guide. https://www.ipec-europe.org/guidelines.html.

[69] Polyplasdone™ Crospovidone Superdisintegrants Product Overview. http://www.ashland.com/Ashland/Static/Documents/ASI/PC_11319_Polyplasdone_Overview.pdf.

[70] Wu Y., Levons J., Narang A.S., Raghavan K., Rao V.M. (2011). Reactive impurities in excipients: Profiling, identification and mitigation of drug-excipient incompatibility. AAPS PharmSciTech.

[71] Rowe R.C., Sheskey P.J., Quinn M.E. (2009). Crospovidone. Handbook of Pharmaceutical Excipients.

[72] Boetzel R., Schlingemann J., Hickert S., Korn C., Kocks G., Luck B., Blom G., Harrison M., François M., Allain L. (2022). A nitrite excipient database: A useful tool to support N-nitrosamine risk assessments for drug products. J. Pharm. Sci..

[73] Rowe R.C., Sheskey P.J., Quinn M.E. (2009). Mannitol. Handbook of Pharmaceutical Excipients.

[74] Pearlitol^®^ 200 SD The Ultimate Mannitol for DC Tablets. http://www.roquette-pharma.com/brochures/17/visio.html.

[75] Nasr N.E.H., Metwaly M.G., Ahmed E.O., Fares A.R., ElMeshad A.N. (2021). Investigating the root cause of N-nitrosodimethylamine formation in metformin pharmaceutical products. Expert Opin. Drug Saf..

[76] FDA-U.S. Food & Drug Administration (2021). Control of Nitrosamine Impurities in Human Drugs Guidance for Industry. https://www.fda.gov/media/141720/download.

